# Molecular Factors and Pathways of Hepatotoxicity Associated with HIV/SARS-CoV-2 Protease Inhibitors

**DOI:** 10.3390/ijms24097938

**Published:** 2023-04-27

**Authors:** Cheng Ji

**Affiliations:** Research Center for Liver Disease, GI/Liver Division, Department of Medicine, Keck School of Medicine of USC, University of Southern California, Los Angeles, CA 90089, USA; chengji@usc.edu

**Keywords:** anti-AIDS/COVID-19 drugs, side effects, substance use disorders (SUD), alcohol use disorder (AUD), ER/Golgi stress, unfolded protein response, liver disease, safer drug design

## Abstract

Antiviral protease inhibitors are peptidomimetic molecules that block the active catalytic center of viral proteases and, thereby, prevent the cleavage of viral polyprotein precursors into maturation. They continue to be a key class of antiviral drugs that can be used either as boosters for other classes of antivirals or as major components of current regimens in therapies for the treatment of infections with human immunodeficiency virus (HIV) and severe acute respiratory syndrome coronavirus 2 (SARS-CoV-2). However, sustained/lifelong treatment with the drugs or drugs combined with other substance(s) often leads to severe hepatic side effects such as lipid abnormalities, insulin resistance, and hepatotoxicity. The underlying pathogenic mechanisms are not fully known and are under continuous investigation. This review focuses on the general as well as specific molecular mechanisms of the protease inhibitor-induced hepatotoxicity involving transporter proteins, apolipoprotein B, cytochrome P450 isozymes, insulin-receptor substrate 1, Akt/PKB signaling, lipogenic factors, UDP-glucuronosyltransferase, pregnane X receptor, hepatocyte nuclear factor 4α, reactive oxygen species, inflammatory cytokines, off-target proteases, and small GTPase Rab proteins related to ER-Golgi trafficking, organelle stress, and liver injury. Potential pharmaceutical/therapeutic solutions to antiviral drug-induced hepatic side effects are also discussed.

## 1. Overview of Antiviral Protease Inhibitors and Side Effects on the Liver

Viral infections by the human immunodeficiency virus (HIV) and/or severe acute respiratory syndrome coronavirus 2 (SARS-CoV-2) have collectively sickened hundreds of millions of people globally and demand the continuous development and repeated use of antiviral medications. While effective anti-HIV vaccines are yet to be developed, the anti-retroviral treatment (ART) introduced in 1996 for HIV infection has been used currently in about 25.5 million people living with HIV (PLWH) and has improved the quality of life as well as the lifespan of these people [1,2,3,4]. ART is composed of a couple of classes of chemical inhibitors that interfere with vital stages in the life cycle of the virus [4]. The inhibitors include nucleoside reverse-transcriptase inhibitors (NRTIs), non-nucleoside reverse-transcriptase inhibitors (NNRTIs), protease inhibitors (PIs), integrase strand transfer inhibitors (INSTIs), and entry inhibitors. Two NRTIs were generally used in association with another class, such as PI or INSTI, which can be modified based on toxicity and induced resistance. PIs are peptidomimetic molecules that were developed by a rational drug design based upon X-Ray crystallographic studies defining the three-dimensional molecular structure of HIV aspartic protease. PIs block the catalytic site of the HIV protease that is responsible for the cleavage of polyprotein precursors into the production of all viral enzymes and structural proteins necessary to produce mature and virulent virions [4]. FDA-approved PI drugs included amprenavir (APV), atazanavir (ATV), darunavir (DRV), fosamprenavir (FPV), indinavir (IDV), lopinavir (LPV), nelfinavir (NFV), ritonavir (RTV), saquinavir (SQV), and tipranavir (TPV). Presently, SQV and APV are no longer marketed, and RTV and DRV are the most frequently used for clinical treatment.

**Figure 1:**
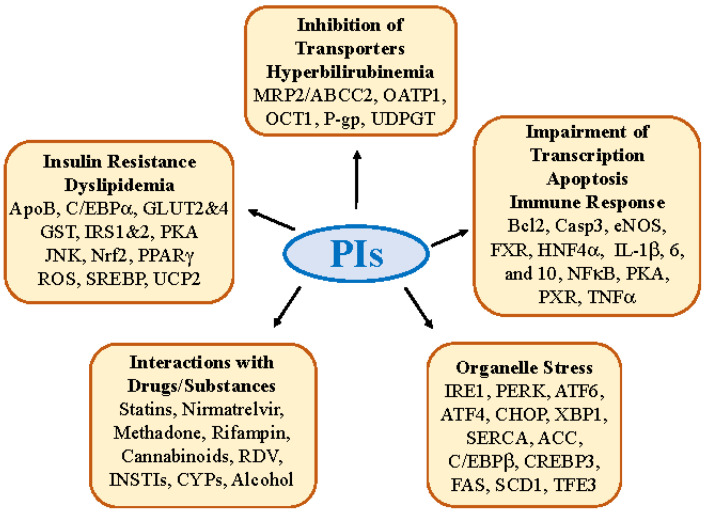
Adverse effects of antiviral protease inhibitors (PIs) on the liver. ApoB, apolipoprotein B; ACC, acetyl-CoA carboxylase; ATF 4 and 6, activating transcription factor 4 and 6; Bcl2, B-cell lymphoma-2; Casp3, caspase 3; C/EBP α and β, CCAAT/enhancer-binding protein α and β; CHOP, C/EBP homology protein 10; CREBP3, CAMP responsive element binding protein 3; CYPs, cytochrome P450 enzymes; eNOS, endothelial nitric oxide synthase 3; FAS, fatty acid synthase; FXR, farnesoid X receptor; GLUT2&4, glucose transporter 2 and 4; GST, glutathione-S-transferase; HNF4α, hepatocyte nuclear factor 4α; HO1, heme oxygenase-1; IL-1β, 6, and 10, interleukin 1β, 6, and 10; INSTIs, integrase strand transfer inhibitors; IRS1&2, insulin receptor substrate 1 and 2; IRE1, inositol requiring enzyme 1; JNK, c-JUN N-terminal kinase; MRP2/ABCC2, canalicular efflux transporter multidrug resistance-associated protein 2; NFκB, nuclear factor κ B; Nrf2, nuclear erythroid-derived factor 2; OATP1, organic anion transporting polypeptide 1; OCT1, human organic cation transporter 1; PERK, PKR-like ER- localized eIF2α kinase; P-gp, P-glycoprotein; PKA, protein kinase B; PPARγ, peroxisome proliferator- activating receptor γ; PXR, pregnane X receptor; RDV, remdesivir; ROS, reactive oxygen species; SCD1, stearoyl-CoA desaturase 1; SERCA, sarco/endoplamic reticulum Ca2+-ATPase; SREBP, sterol regulatory element binding protein; TFE3, transcription factor for immunoglobulin heavy-chain enhancer 3; TNFα, tumor necrosis factor α; UCP2, mitochondrial uncoupling protein 2; UDPGT, uridine-diphosphoglucuronic glucuronosyltransferase; XBP1, X-box binding protein 1.

HIV PIs have been on the World Health Organization’s list of essential medicines for ART. During the outbreak of Coronavirus Disease-19 (COVID-19) and emergency needs, PIs have attracted much attention from the public and become the backbone of antiviral regimens. At the time when there were no effective antiviral treatments for the coronavirus SARS-CoV-2 infection, LPV-RTV was among the earliest drugs used in randomized, controlled, and open-label trials in hospitalized patients with severe COVID-19. In the middle of the COVID-19 pandemic, RTV-boosted nirmatrelvir (Paxlovid) was found to be the most effective against the protease M^pro^ of SARS-CoV-2 and its variants or subvariants [5,6,7]. Paxlovid is now widely used in both at-home and hospitalized patients with COVID-19, and there is a trend that PIs could be used regularly and repeatedly due to emerging coronavirus variants and frequent rebounds of the viral infection. Liver injuries were observed when the anti-SARS-CoV-2 PIs were used for more than five days or in combination with other drugs or substances [6,7,8]. In anti-HIV therapies, despite the fact that the currently preferred regimens are integrase inhibitors, PIs are still needed either alone or as boosters for newly developed INSTIs, including raltegravir, elvitegravir, bictegravir, and dolutegravir (DTG). However, these unprecedented massive, intense, and/or prolonged antiviral medications with PIs have complications and side effects. There are numerous reports indicating that ART with PI drugs singly or in combination increases the risk of comorbidities, including dyslipidemia, insulin resistance, lipodystrophy/lipoatrophy, pancreatitis, cerebrovascular diseases, immune restoration disease, and hepatotoxicity/liver dysfunctions (e.g., coagulopathy, encephalopathy, ascites, hyperbilirubinemia, fibrosis, and cirrhosis) [3,8,9,10,11,12,13]. While some side effects of PI drugs are manageable, some can be very serious and fatal. For instance, before the introduction of ART, HIV/AIDS patients could develop dyslipidemia characterized by isolated elevation of triglycerides, total cholesterol, and other proatherogenic lipids [11,12]. With the advent of ART, especially with the combined use of PI drugs, hepatotoxicity has emerged as a major non-AIDS–related cause of death among HIV/AIDS patients [9,10,11,12,13]. All the PIs have been associated with transient and usually asymptomatic elevations in serum aminotransferase levels and ATV and IDV with mild-to-moderate elevations in indirect and total bilirubin concentrations. Without substance use disorders (SUD), PIs are rare causes of clinically acute liver injury. With SUD, which is often associated with alcohol use disorders (AUD) and psychiatric comorbidities, PI-induced hepatic injuries become much more severe and chronic [14,15,16]. Increased rates of fatty liver disease have been reported in HIV or SARS-CoV-2 mono-infected subjects, mostly with persistently elevated liver enzymes [3,6,9,12]. Dyslipidemia is much more prone to developing hepatic steatohepatitis and cirrhosis [11,12,13]. The molecular factors and mechanisms underlying PI-associated hepatotoxicity are not fully understood and are under intense investigation. New advances in the investigations are summarized and discussed in this review (Figure 1). For general knowledge, the phenotypes of side effects, or clinical observations related to antiviral PIs, readers are referred to other reviews [4,10,11,12].

## 2. Molecular Factors and Pathways Involved in PI-induced Hepatotoxicity

### 2.1. Insulin Resistance, Dyslipidemia and Lipodystrophy

Insulin resistance, dyslipidemia, and lipodystrophy are characteristic side effects that are developed early in HIV/AIDS patients treated with LPV, RTV, and SQV. The side effects are mild in patients treated with recently developed PIs, such as darunavir. Insulin resistance decreases the utilization of insulin-mediated total body glucose in skeletal muscle, increases basal lipolysis in the adipose tissue, and increases hepatic glucose production and the secretion of very low-density lipoproteins in the liver [17,18,19,20]. PI-induced insulin resistance contributes to overall body dyslipidemia with high levels of total cholesterol, low-density lipoprotein, very low-density lipoprotein, and triglycerides. These lipid abnormalities result in the lipodystrophy syndrome characteristic of metabolic disturbances and apparent changes in fat tissue distribution with fat loss in all depots, excluding the abdominal region [20,21,22,23,24]. There are a few molecular factors and pathways that have been identified as contributing to PI-induced insulin resistance, which includes the inhibition of glucose transporters, impaired insulin signaling, and altered lipogenic regulators and mitochondrial function (Figure 1, Table 1).

First, HIV PIs selectively inhibit the transport function of insulin-sensitive glucose transporter GLUT4. The effects of RTV on glucose tolerance were first tested in transgenic mice lacking GLUT4 (G4KO), which determined the specific contribution of PI-mediated GLUT inhibition and altered glucose homeostasis [25]. IDV was later shown to inhibit GLUT4 acutely and reversibly in fat and muscle [26]. IDV inhibited insulin-stimulated glucose uptake at pharmacologically relevant drug levels in cultured adipocytes [27], which provided a direct explanation for peripheral insulin resistance. NFV was also reported to suppress the function of GLUT4, diminishing insulin-stimulated glucose uptake in the liver and adipose tissues [28]. In vitro findings with IDV correlated with the acute and reversible induction of insulin resistance in vivo both in rodents and in HIV-negative volunteers [29,30]. Thus, GLUT4 inhibition and impaired glucose transportation can be the primary mechanism leading to glucose tolerance and adaptations in muscle and adipocytes, as well as a rise in glucose uptake and lipid synthesis in the liver [31,32]. The GLUT4 inhibition indirectly contributed to hepatic insulin resistance through the hypothalamic control of hepatic glucose synthesis [33]. HIV PIs directly interfere with other molecular pathways that influence insulin release from the pancreas and insulin signaling in the liver. Further, RTV, NFV, and SQV not only induce peripheral insulin resistance but also impair glucose-stimulated insulin secretion from beta cells [34]. Impaired insulin signaling has also been observed in cultured HepG2 cells treated with IDV [35]. IDV interfered with insulin stimulation on the insulin-receptor substrate (IRS)-1-phosphorylation, the association of phosphatidylinositol 3-kinase with IRS1-1, and Akt-Thr308-phosphorylation of AKT/PKA signaling in the liver cells [27,36]. IDV also had direct in vitro effects on GLUT2: a hepatic facilitative glucose transporter that plays an important role in the normal function of the hepatoportal glucose sensor [37]. The HIV PI-induced impairment of insulin signaling and increased hepatic lipid production might further stimulate gluconeogenesis and activate PKCε and JNK1 (c-JUN N-terminal kinase), which can interfere with the tyrosine phosphorylation of IRS-1 and IRS-2 and alter the ability of insulin to activate glycogen synthase [38,39].

Second, apolipoprotein B (ApoB), the principal protein component of triglyceride and cholesterol-rich plasma lipoproteins, was identified to be a lipogenic factor connecting the altered metabolism and pathogenesis of PI-associated lipodystrophy. In cultured human/rat hepatoma cells and primary hepatocytes from transgenic mice, HIV PIs inhibited the proteasomal degradation of nascent ApoB, inhibited the synthesis of the cholesteryl-ester and activity of the microsomal triglyceride transfer-protein, and affected the secretion of ApoB-containing lipoprotein particles [40]. HIV PIs also have effects on other lipogenic factors. PIs affect cellular levels of the peroxisome proliferator-activating receptor (PPAR) γ and CCAAT/enhancer-binding protein (C/EBP) α, both of which are important in preadipocyte differentiation into mature adipocytes and lipid homeostasis [41]. PIs suppress the breakdown of the nuclear form of sterol regulatory element binding proteins (nSREBP) in both the liver and adipose tissues. The hepatic accumulation of nSREBP results in increased fatty acid and cholesterol biosynthesis, whereas nSREBP accumulation in adipose tissue causes lipodystrophy, reduces leptin expression, and promotes insulin resistance [42,43].

Third, evidence supporting the role of PIs in inducing oxidative stress has been accumulated in many model systems, and oxidative stress often leads to hepatic insulin resistance and lipodystrophy [44,45,46,47,48]. NFV increased the significant generation of reactive oxygen species (ROS) and mitochondrial uncoupling protein 2 (UCP2) and decreased cellular levels of glutathione and ATP in HIV-infected patients [44]. Treatment with thymoquinone (TQ), which has antioxidant, anti-inflammatory, and anti-cytotoxic activities, significantly ameliorated the oxidative effects of NFV [45], suggesting the direct role of NFV in inducing oxidative stress. ATV was reported to interfere with the function of cytochromes P450 (CYPs), increasing lipid peroxidation and ROS and decreasing the passive transport of PI from the blood to the liver in a rat model [46]. The RTV treatment of Huh-7.5 (human hepatoma cells) or Hepa RG (hepatic progenitor cells) showed increased oxidative cellular stress even after only six hours of exposure [47]. Both PIs and HIV infection seemed to contribute to oxidative stress. Transgenic rats expressing HIV-1 had enhanced hepatic genomic changes relating to oxidative/nitrosative stress and lipogenesis and treating animals with a clinically used regimen containing ATV and RTV deteriorated the oxidative stress and lipid accumulation [48]. Magnesium supplementation increased the expression of the key redox regulator Nrf2 (nuclear erythroid-derived factor 2), HO1 (heme oxygenase-1), and GST (glutathione-S-transferase), as well as reducing the expression of the lipogenic gene SREBP-1, which completely prevented the HIV PI-induced oxidative stress and lipid accumulation. PIs also had an oxidative impact on Kupffer cells, as the most abundant human liver macrophages, playing a critical role in maintaining hepatic and whole-body cholesterol homeostasis [49]. RTV has been shown to inhibit cholesterol efflux from macrophage-derived foam cells and increase lipid peroxidation and oxidative stress in intracellular mitochondria, resulting in increased cholesteryl ester transfer proteins that decreased levels of circulating high-density lipoprotein-cholesterol and increased levels of very low-density lipoprotein-cholesterol [49,50]. Thus, mitochondrial dysfunction caused by PI-induced oxidative stress promotes fatty infiltration in the liver and muscle, further exacerbating insulin resistance and lipodystrophy [51].

### 2.2. Hepatic Transporter Inhibition and Hyperbilirubinemia

Liver transporters play an essential role in the cellular uptake, excretion, and elimination of various xenobiotic drugs and in drug–drug interactions. IDV, NFV, RTV, and SQV are potent inhibitors for the human organic cation transporter (OCT) 1 [52]. Some of the PIs are also poor substrates for OCT1 and potentially inhibit the uptake and metabolism of other cationic drugs, leading to hepatotoxic drug–drug interactions (Table 1). In rat and human hepatocytes, the majority of PIs were reported to inhibit the canalicular efflux transporter multidrug resistance-associated protein 2 (MRP2/ABCC2) [53,54]. RTV and SQV were shown to directly inhibit the bile acid transport MRP2, resulting in the biliary accumulation of the fluorescent substrate 5(6)-carboxy-2′,7′-dichlorofluorescein [55]. The organic anion transporting polypeptide (OATP)-1B1 and OATP1B3 are expressed on the sinusoidal membrane of hepatocytes, playing a key role in the hepatic disposition of several HIV PIs [56]. However, some HIV PIs, such as LPV, are also OATP1B inhibitors causing the accumulation of OATP1B substrate fexofenadine resulting in clinically relevant drug–drug interactions [57,58]. The inhibition of hepatic transporters by PIs and subsequent drug–drug-interactions often impair liver bilirubin uptake and metabolism, leading to elevations of serum bilirubin (hyperbilirubinemia) [59]. In addition, HIV PIs may directly act on enzymes of hepatocytes that conjugate bilirubin into glucuronic acid. Elevations in serum-unconjugated bilirubin were reported to be associated with IDV treatment, and IDV was found to directly inhibit the bilirubin-conjugating activity catalyzed by uridine-diphosphoglucuronic glucuronosyltransferase (UDPGT) [60]. ATV-induced hyperbilirubinemia has often been reported [61,62,63,64,65]. In a study involving a population of 2400 patients, as many as one-third of individuals taking ATV developed hyperbilirubinemia and presented hepatotoxicity manifested by transaminase flares [61]. The adverse effects of PIs on serum bilirubin are also associated with Gilbert’s syndrome: a frequent genetic conjugation abnormality associated with UDPGT alleles. In ATV treatment, the risk of severe hyperbilirubinemia could be specifically associated with the genetic variant UDPGT1A1*28 [62,63,64,65], whereas in IDV treatment, the risk was associated with UDPGT1A3 and UDPGT1A7 genes in addition to UDPGT1A1*28 [66].

### 2.3. Impairment in Hepatic Transcription, Apoptosis, and Immune Response

The HIV PI treatment-associated dyslipidemia may result from hepatic transcriptional changes in lipogenic genes such as SREBP and fatty acid synthase. The bile acid-activated nuclear farnesoid X receptor (FXR) was reported to regulate the lipogenic genes, as well as to counter-regulate the expression/activity of CD36 on macrophages in rodent models treated with RTV [67]. The FXR agonist, chenodeoxycholic acid, protected against the development of dyslipidemia and vascular injury induced by RTV. In addition to FXR, the HIV PIs impair transcriptional functions of the pregnane X receptor (PXR) and hepatocyte nuclear factor 4α (HNF4α). For instance, PXR was reported to potentiate RTV hepatotoxicity in all participants of multiple clinical studies and in PXR-humanized mouse models treated with rifampicin or efavirenz that activate PXR [68]. Mechanistically, PXR interfered with CYP3A4 activities resulting in oxidative and cellular stress. PXR also has an impact on RTV-impaired hepatic glucose metabolism and hyperglycemia. This involves HNF4α and GLUT2. In both HepG2 cells and primary mouse hepatocytes, PXR agonists atorvastatin and rifampicin reduced HNF4α, GLUT2 expression, and glucose uptake and utilization [69]. Silencing the *PXR* gene upregulated HNF4*α* and GLUT2 expression, and PXR overexpression downregulated GLUT2 and HNF4*α* expression [69]. The mechanistic involvement of the PXR-HNF4*α* pathway was further confirmed when the long noncoding antisense RNA 1 of HNF4*α* was recently demonstrated to modulate the expression of PXR and CYP3A4 and increase cytotoxic lactate dehydrogenase and reactive oxygen species in Huh7 and HepG2 cells [70].

PI-impaired transcription and lipid synthesis can lead to other hepatotoxic changes. Morphological and ultrastructural changes, including chromatin margination, mitochondrial cristae disappearance, karyopyknosis, and cytoplasmic vacuolization, were observed in hepatocytes derived from RTV-treated mice [71]. HIV PIs were reported to induce cell cycle arrest in G0/G1 and inhibit hepatocyte proliferation by modulating the NFκB/pAkt signaling molecules [72,73]. RTV increased the expression of Bax, reduced the expression of Bcl-2, activated caspase 3 and caspase 8, and ultimately led to cell death [72,73,74]. The apoptosis impaired by HIV PIs could have further adverse effects on hepatic immune responses, including increased inflammatory cytokines such as TNF-α, IL-1β, and IL-6 and decreased anti-inflammatory mediators such as IL-10 [75,76]. Isoflavones such as formononetin and biochanin A were reported to alleviate RTV-induced hepatotoxicity through attenuating Bax, caspase-3, NFκB, and eNOS activation and reducing Bcl_2_ and pAkt levels [73].

### 2.4. Molecular Interactions of PIs with Other Drugs/Substances

It has been well reported that a significant portion of HIV PIs-induced hepatotoxicity results from their interactions with other drugs used in ART and in co-infections with hepatitis virus or coronavirus [3,14,77,78,79,80,81,82]. One common mechanism involves the cytochrome P450 enzymes, as the majority of HIV PIs drugs were oxidized/metabolized by CYP450, particularly the CYP3A isozyme predominantly present in the liver (Table 1). HIV PIs can exert hepatotoxic effects by acting as competitive inhibitors, inducers, and/or substrates of the CYP450 enzyme system. For instance, RTV boosts the efficiency of nonpeptidic PI-tipranavir for the treatment of drug-resistant HIV infection. Both cannabinoid and protease inhibitors, such as IDV and NFV, share CYP450 metabolic pathways, and their interactions perturbated the pharmacokinetics of PIs as well as the hepatic endocannabinoid system leading to hepatic inflammation and chronic liver damage [79]. Severe hepatotoxicity was observed due to the RTV inhibition of CYP3A4 that metabolizes TPV [83,84,85]. Similarly, the hepatitis C virus (HCV)-PIs boceprevir and telaprevir were both, to different extents, inhibitors of CYP3A and boosted HIV PIs such as ATV, DRV, and LPV causing elevations of aminotransferase and hepatic injury [86]. Antifungal azoles inhibit the lanosterol 14-α-demethylase enzyme (P45014DM) that is responsible for cholesterol biosynthesis and are potentially hepatotoxic drugs commonly used by HIV-infected patients receiving booster protease inhibitors [87]. The antituberculosis drug rifampicin used in HIV patients has the greatest effects on the expression of CYP3A4 in the liver, which often compromises HIV treatment with PIs such as SQV and RTV [88,89,90,91].

In addition to CYP450, drug uptake transporters such as OATP1B1 and drug efflux transporters such as P-glycoprotein (P-gp) and breast cancer resistance protein (BCRP) are other molecular factors involved in HIV PI–drug interactions resulting in liver injury [53,92,93,94]. The HMG-CoA reductase inhibitors (also known as statins) are a class of drugs widely prescribed for the treatment of hypercholesterolemia and the prevention of cardiovascular morbidity and mortality. Since they are extensively metabolized by CYP3A4 and CYP3A5, statins show a high potency for drug–drug interactions with potent CYP3A inhibitors such as RTV/cobicistat-boosted HIV integrase inhibitor elvitegravir or HCV PIs (e.g., telaprevir and boceprevir) [95]. Non-CYP3A-dependent statins were also affected to a lesser extent when co-administered with HIV or HCV PIs, mainly through their interaction with OATP1B1. In a similar fashion to the medicines used in hepatitis co-infection, RTV, DRV, and LPV interact with multiple drugs, including amoxicillin, interferon, ribavirin, oseltamivir, molnupiravir, and nirmatrelvir, which are used in SARS CoV-2/COVID-19 treatments. RTV combined with LPV was reported to interfere with gamma-glutamyltransferase in cholangiocytes, highly expressing the angiotensin-converting enzyme 2 receptors (ACE2) and leading to abnormalities in the total bilirubin and elevated ALT/AST levels greater than five times [96,97]. Similar biliary and hepatic injuries could occur in patients treated with RTV in combination with molnupiravir or nirmatrelvir for more than five days [6,98,99]. In addition, PLWH under ART may have substance use disorders (SUD) with fentanyl, methadone, ketamine, or cannabinoids that are metabolized by cytochrome P-450 enzymes. HIV PIs interact with these substances by impacting their uptake, transport, and intrinsic hepatic clearances. Cytotoxic high levels of reactive metabolite formation, impaired methadone demethylation, and irreversible covalent binding to microsomal proteins were observed in hepatocytes and macrophages from HIV patients with SUD [100,101,102,103,104].

## 3. Antiviral PI-Induced Organelle Stress Pathways and Specific Off-Targets

### 3.1. Hepatic ER and Golgi Stress Response

**Figure 2:**
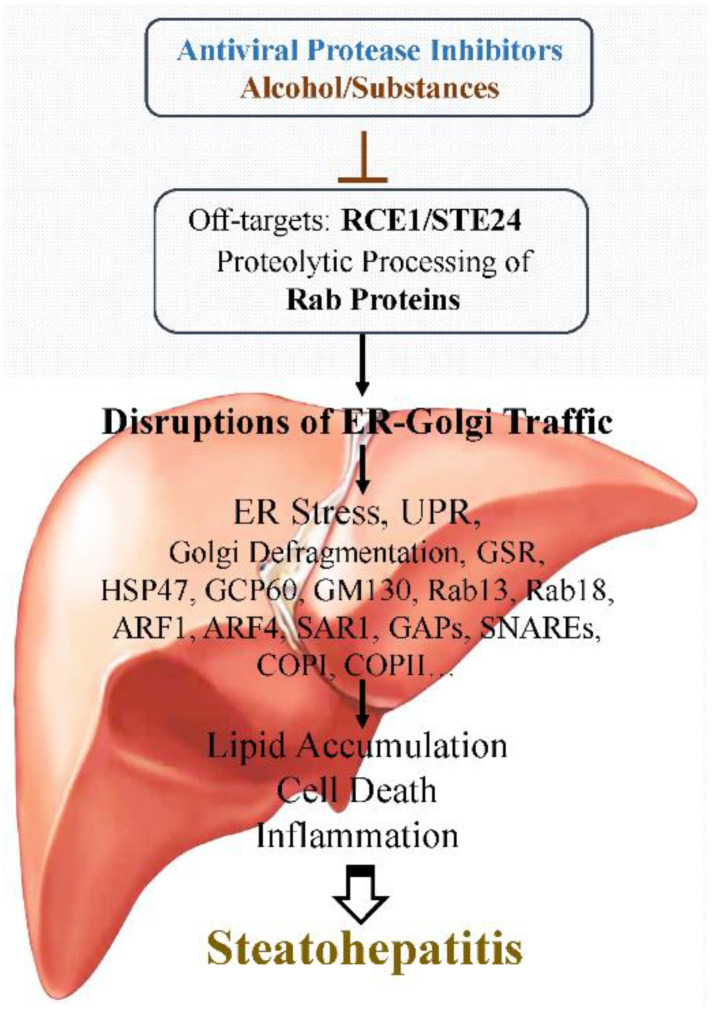
Mechanisms of antiviral protease inhibitor-induced organelle stress and liver injury. ARF1/4, ADP-ribosylation factor 1/4; COP I/II, coat protein complex I/II; GAPs, GTPase-activating proteins; GCP60, Golgi resident protein 60; GM130, Golgi matrix protein; GSR, Golgi stress response; HSP47, heat shock protein 47; Rab, small GTPase binding protein; RCE1, the Ras converting CaaX endopeptidase 1; SAR1, secretion-associated RAS-related; SNAREs, tail-anchored membrane fusion proteins; STE24, ER-associated CaaX protease; UPR, unfolded protein response.

Two intracellular organelles, the endoplasmic reticulum (ER) and Golgi apparatus are essential for lipid and protein homeostasis, and disrupting their functions triggers stress responses and leads to metabolic disorders and hepatic pathologies such as steatohepatitis and liver fibrosis/cirrhosis [105,106,107,108]. (Figure 1 and Figure 2). In ER, stress initially triggers a protective unfolded protein response (UPR) [109,110], which involves three ER stress sensors: IRE1 (inositol requiring enzyme 1), PERK (PKR-like ER-localized eIF2α kinase), and ATF6 (activating transcription factor 6), to restore ER homeostasis and minimize potential stress caused injuries. However, prolonged UPR, such as under sustained antiviral medications, alcohol consumption, or SUD, alters the expression of key metabolic transcriptional regulators, including GRP78/BiP (immunoglobulin heavy chain-binding protein), C/EBPβ (CCAAT-enhancer-binding protein β), C/EBPδ, CHOP (C/EBP homology protein 10), XBP1 (X-box binding protein 1)/sXbp1 (spliced active Xbp1 or Xbp-1s), ATF4 (transcription factor-4), Nrf2, and PPARγ, resulting in cell death and the development of hepatic steatosis that involve many lipogenic factors including SREPBs, ACC (acetyl-CoA carboxylase), VMP1 (vacuole membrane protein 1), DGAT2 (diacylglycerol acyltransferase-2), ACL (ATP citrate lyase), FAS (fatty acid synthase), and SCD1 (stearoyl-CoA desaturase) [105,111,112]. Similar to the ER, Golgi can also be stressed under prolonged alcohol and/or antiviral medications, which initiates the Golgi stress response (GSR). GSR involves a few regulatory factors/pathways: TFE3 (transcription factor for immunoglobulin heavy-chain enhancer 3), the CREB3-ARF4 pathway (CREBP3, CAMP responsive element binding protein 3; ARF4, ADP ribosylation factor 4), and HSP47 (heat shock protein 47 [108,113]. TFE3 regulates the transcriptional activation of genes encoding vesicular trafficking components or Golgi resident enzymes. The CREB3-ARF4 pathway involves both ER and Golgi, in which ARF4 is a member of the small GTPase family that regulates Golgi-to-ER vesicular trafficking. HSP47 is an ER chaperone, which binds to IRE1, releases the ER master chaperone BiP, and protects cells against Golgi stress and cell death.

### 3.2. PI-induced Organelle Stress Response

HIV PIs have long been known to induce organelle stress that, in general, links to the dysregulation of lipid metabolism in the liver [77,114,115,116,117,118]. RTV-boosted LPV or DRV was reported to induce the dilatation of ER and dispersed/fragmented Golgi apparatus [115,119,120,121]. RTV increased the activity of complexes I and IV, with simultaneous uncoupling and the inhibition of complex V, contributing to mitochondrial dysregulation and cell death [47,122,123]. The phosphorylation of eIF2α and activation of ATF4 was increased in the liver of mice treated with NFV [124]. In mouse and human primary hepatocytes, LPV/RTV treatment combined with alcohol-inhibited sarcoplasmic reticulum Ca^2+^-ATPase (SERCA) expression, which disturbed ER calcium homeostasis, exacerbating ER stress and resulted in excessive liver cell death [115,121]. The three canonical UPR branches, IRE, PERK, and ATF6, were reported as being differentially expressed in hepatocytes in response to RTV-LPV [119]. The ATF6 branch and its downstream factors were inactivated, while the other two branches of UPR, IRE1, represented by the expression of sXbp1 mRNA, and PERK and represented by the expression of CHOP, were upregulated. These observations are interesting because the activation of ATF6 is known to require trafficking from ER to Golgi for proteolytic processing and involves both ER and Golgi. In fact, the co-localization of ATF6 and the Golgi matrix protein GM130 was lower in liver cells treated with PIs than in cells treated with tunicamycin, which induced only ER stress [119]. Golgi stress markers, GCP60 and HSP47, were also increased remarkably in the hepatocytes treated with PIs. The inhibition of ATF6 nuclear translocation and subsequent induction of Golgi stress by the anti-HIV drugs were quite specific as the ER stress-inducing agents, tunicamycin and thapsigagin, did not exert similar effects, and the knockdown of TFE3 by RNA interference worsened PI-induced cell death [119,125].

The inhibitory effects of PIs on ATF6 processing and activation reflect interference with the inter-organellar communication between ER and Golgi. The Golgi is part of the cellular endomembrane system, which receives secretory and membrane proteins from the ER and delivers them to various destinations via the formation of an ER–Golgi interface and bidirectional membrane trafficking between the two organelles. This trafficking requires the coat protein complex I (COPI) and COPII [126,127,128,129]. COPI participates in the retrograde route from Golgi to ER, and COPII participates in the anterograde route from ER to Golgi. The initiation of the retrograde route requires COPI, ADP-ribosylation factor 1 (ARF1), GTPases, and other co-factors [129]. The initiation of the anterograde route requires COPII, secretion-associated RAS-related 1 (SAR1), GTPases, and GTPase-activating proteins (GAPs). Both routes are required for the structural and functional integrity of the two organelles. However, the integrity of the anterograde route depends on the retrograde route, which ensures not only the retrieval of resident proteins from the ER but also facilitates the recycling of lipids and traffic machinery [129,130,131]. In RTV-LPV-treated liver cells, COPII was found to be aggregated, whereas Brefeldin A (BFA), which is known to inhibit the retrograde route [119,125], did not prevent PI-induced COPII aggregation, suggesting that RTV-LPV interfered with COPII-mediated anterograde trafficking. RTV-LPV also inhibited the distribution of Golgi-resident enzyme mannosidase alpha class 2A member 1 (MAN2A1) to Golgi. In parallel to the impaired ER-Golgi trafficking and ER/Golgi stress response, marked Golgi fragmentation, fat accumulation, and cell death were observed in the hepatocytes from mice treated with the PIs, dexamethasone (DEX), and/or remdesivir (RDV) [115,119,125,126]. Moreover, the severity of Golgi fragmentation in response to other PIs, including TFV, EFV, DTG, DRV, APV, and NFV, was well correlated with downstream hepatic injury [119,125].

### 3.3. Specific Off-Targets of PIs Linking to ER-Golgi Trafficking and Dyslipidemia

The precise mechanisms for lipid abnormality and lipodystrophy often seen in AIDS patients under ART are still not fully understood. It likely results from the off-targeting effects of HIV aspartyl PIs (Figure 2). HIV PIs were reported to block ZMPSTE24 (the human ortholog of STE24 protease) and interfere with the endoproteolytic processing of the mammalian farnesylated proteins, prelamin A, which alter the maturation and stability of lamin A and the nuclear localization of SREBP-1 [132]. The cholesterol content of membranes, cells, and blood can be controlled by the integral membrane zinc metalloprotease ZMPSTE24 which resides in both the ER membrane and the inner nuclear membrane [133]. HIV-PIs, including LPV, RTV, and TPV, could directly block the enzymatic activity of purified Ste24p and the yeast ortholog of ZMPSTE24 [134,135]. Newer HIV PIs, such as DRV, with less capability to induce ER stress, do not inhibit ZMPSTE24 or lead to an accumulation of farnesyl-prelamin A in cells [136]. Recent evidence implicates Ste24 as a key factor in several ER processes, including the UPR, the removal of misfolded proteins from the translocon, and lipogenic abnormalities in the liver, muscle, and adipose tissues [137,138]. In addition, mutations in ZMPSTE24 diminish its activity, giving rise to cell senescence and calcification, excess visceral abdominal fat, and genetic diseases of accelerated aging (progerias) that can be seen in HIV patients under PI treatment [139,140]. Thus, off-targeting the enzymatic activity of ZMPSTE24 and subsequent blocking prelamin A processing likely contribute to lipodystrophy in individuals undergoing HIV-PI treatment.

Another potential host off-target of the HIV PIs is the Ras converting *CaaX* endopeptidase 1 (RCE1), which is a glutamyl protease that can be classified as a member of the zinc metalloproteinase family [141,142]. RCE1 was initially identified through the total RNA-sequencing of RNA from HepG2 cells treated with RTV and LPV [125]. The levels of the RCE1 protein were inhibited not only in cultured HepG2 but also in primary hepatocytes and in the liver of mice treated with the anti-HIV drugs. Neither Rce1 transcription nor the RCE1 protein level was inhibited by Brefeldin A, which also induced organelle stress in the liver cells, suggesting that RCE1 could be specifically inhibited by the anti-HIV drugs. In addition, knocking down Rce1 with RNA interference increased RTV and LPV-induced cell death and the subsequent expression of Golgi stress response markers, TFE3, HSP47, and GCP60, in both primary hepatocytes and mouse liver, and deteriorated alcohol-induced ALT and fatty liver injury in animal models [125,126]. Further, the HIV PI-induced effects on RCE1 were accompanied by the inhibition of two potential substrates of RCE1, the small GTP binding proteins Rab13 and Rab18. Rab13 and Rab18 are special GTPases that contain the *CaaX motif* (where C is cysteine, A is an aliphatic amino acid, and X is any amino acid) [142]. Rab proteins are known to play an important role in regulating/coordinating the ER-Golgi traffic, which involves many effectors such as vesicle tethers, SNAREs, membranes, and motor proteins [143,144]. The disruption of the coordination has been shown to cause ER/Golgi stress, excessive lipogenesis, and abnormal lipid turnover [145,146,147]. The HIV PI and alcohol-induced Golgi fragmentation, Golgi stress response, and cell death could be reduced in the primary human hepatocytes overexpressing Rab13 [125,126]. While the direct inhibition of RCE1 enzyme activity by the HIV PIs needs to be tested further, these pieces of evidence support the host protease RCE1 as an off-target of HIV PIs.

## 4. Potential Therapeutic/Pharmaceutical Solutions for Antiviral PIs Associated with Liver Injury

### 4.1. Targeting Insulin Resistance, Cellular Stress and Dyslipidemia

Natural compounds have been used as potential therapeutic agents or as dietary supplementation for antiviral PI-induced deleterious effects on the liver. First, thymoquinone extracted from black seed oil has been shown to protect HIV-infected patients against ATV, NFV, or SQV-induced oxidative stress, glucose intolerance, and the impairment of insulin signaling and lipodystrophy [45,148,149,150,151]. Thymoquinone is beneficial for COVID-19 prevention and cure in patients under anti-SARS-CoV-2 treatment [151]. Second, flavonoids and isoflavones are also natural products possessing anti-inflammatory, antioxidant, and anti-apoptosis activities and can be used to alleviate the side effects of HIV PI drugs. Naringin, a grapefruit-derived flavonoid, reversed weight loss, polydipsia, elevated fasting blood glucose, and reduced levels of fasting plasma insulin, the expression of phosphorylated IRS-1 and Akt proteins, and hepatic glucokinase in rats treated with ATV or SQV [152]. Naringin protected against lipid abnormalities in SQV or DRV-treated rat liver slices and human liver hepatocytes [153]. In addition to HIV PI-induced hepatic injury, naringin protected against NRTI-induced mitochondrial toxicities in zidovudine-treated rats by improving antioxidant enzyme activities, reducing ROS-induced mtDNA damage, and increasing the expression of the complex IV protein [154]. Alcoholic extracts of lotus leaves, rich in flavonoids, have the potential to treat dyslipidemia in rats treated with LPV and RTV [155]. Third, the red clover isoflavones, formononetin, and biochanin A were shown to modulate NFκB/pAkt signaling molecules and protect against RTV-induced hepatotoxicity in in vivo animals [73,156,157]. Some flavonoids, such as quercetin, not only protect against drug-induced hepatotoxicity but are also effective as antiparasitic and anti-HIV/SARS-CoV-2 agents [158].

HIV PI-induced organelle stress can also be a therapeutic target for drug development. However, it can be challenging because, in addition to PI drugs, certain viruses, such as HIV, HBV, HCV, and coronavirus modulate ER stress response or preferentially activate the different pathways of UPR [105,109,112,159,160,161]. For instance, HCV infection activates the ATF6 pathway while blocking the IRE1 pathway and HBV infection, which stimulates both ATF6 and IRE1 signaling but has no effects on PERK signaling [105,159]. Targeting any specific UPR branches could lead to potential side effects on the normal cell functions of other UPR branches and limit the usage of drugs when targeting organelle stress proteins. Despite this challenge, there are a couple of chemical chaperones such as 4-phenylbutyric acid (4-PBA) and tauroursodeoxycholic acid (TUDCA), which can alleviate ER stress by increasing their protein-folding capacity. PBA has a beneficial role in coping with hepatic fat accumulation and lipotoxicity resulting from ER/Golgi stress [162,163]. TUDCA can also alleviate organelle stress stabilizing UPR, reduce oxidative stress and cell death, and decrease inflammation in many in vitro and in vivo models of metabolic syndromes [164,165]. In addition to chemical chaperones, salubrinal, an eIF2α dephosphorylation inhibitor, reduces xenotoxicant-Induced cellular stress and damage [166], which could be useful for the treatment of ER stress–associated liver diseases.

### 4.2. Improving Drug Delivery and Bioavailability

Inefficient drug delivery and poor bioavailability necessitate high drug doses or a combination of drugs with pharmacokinetic enhancers and/or special formulations. For instance, RTV has widely been used as a booster for other antiviral PIs, which often cause severe hepatotoxicity. Improving PI drug pharmaceutical properties for efficient drug delivery or high bioavailability can provide rational solutions to mitigate drug-induced hepatic side effects. There are two potential approaches, the development of prodrugs and the application of nanotechnology for the improvement of pharmacokinetic, delivery, and side effect profiles. Peptide prodrug was reported to improve oral absorption, transport across the intestinal epithelium, mitigate CYP3A4-mediated metabolism and improve solubility profiles of LPV [167,168]. Phosphate prodrugs have been explored to address absorption limited by solubility, and amino acid prodrugs have been shown to improve drug permeability by engaging with active transport mechanisms. For instance, phosphate and amino acid ester prodrugs have improved the oral bioavailability and plasma concentration of ATV by more than fivefold [169,170]. However, the reported prodrugs lack detailed in vivo characterization, and hence, the preclinical or clinical benefits of the prodrugs have yet to be fully determined.

Nanotechnology has provided opportunities to achieve better-targeted PI delivery and enhanced bioavailability. To overcome its intense lipophilicity and extensive metabolism by liver microsomal enzymes CYP3A4, LPV has been prepared with surface-stabilizing nanoparticles, which enhanced bioavailability by more than 3-fold without the coadministration of RTV in a rat model [171]. A children-friendly and flexible solid dosage form of LPV-RTV was also created utilizing novel in situ self-assembly nanoparticles that form granules when in contact with water [172]. PI drug granules were stable under physiological conditions for over 8 h and displayed a nearly 3-fold increase in bioavailability in rats. To effectively reduce the HIV viral load in the brain that often forms an independent viral reservoir resulting in latent infection or debilitating neurological complications, PI drugs were formulated with specific brain-targeting nanocarriers, including polymeric nanoparticles, liposomes, solid lipid nanoparticles, micelles, and macrophage-based nanoparticles. This facilitated drug transport into the brain via endocytic pathways, inhibited the ATP-binding cassette (ABC) transporters expressed at the brain barrier sites, and dramatically increased local bioavailability to the brain [173,174,175,176]. HIV PIs, including ATV, IDV, RTV, and SQV, were successfully delivered across the blood–brain barrier at concentrations that did not cause hepatotoxicity in animal models [174,175,176]. In addition, sustained or prolonged (i.e., 7 to 20 days) release of PI drugs, including ATV, DRV, and RTV, was achieved in vivo using pH-responsive nanoparticles, nanoparticles of mPEG-PCL (methoxy poly (ethylene glycol)-poly (e-caprolactone)), or nanoparticles encapsulating both hydrophilic and hydrophobic PI drugs [176,177,178,179,180].

### 4.3. Designing Safer PIs

The host glutamyl proteases, RCE1 and STE24, belong to the zinc metalloproteinase family [181,182], which are normally not targeted by the HIV-1 aspartyl protease inhibitors. However, recent studies with crystallization and analysis of STE24 revealed that the molecular structure of the STE24 enzyme protein is different from most other zinc metalloprotease members. The internal cavities of the STE24 protease hold several water molecules that enable structural stability and flexibility [183,184]. Such a hydrophilic environment could enhance the tetrahedral coordination of the zinc atoms at the enzyme active site, allowing off-target access by anti-HIV/SARS-CoV-2 protease inhibitors. RCE1 protein may have a molecular structure similar to STE24 as both are *CaaX* endopeptidases. In addition, the chemical properties of the individual antiviral PIs are different, which makes some PIs more prone to off-target access than others resulting in varying degrees of hepatic side effects. For instance, RTV-induced Golgi fragmentation and injury in the liver cells were severer than LPV, and LPV–induced liver damages were severer than DRV [119,125,126]. The overall chemical structures of RTV and LPV are nearly the same, except that the core region of LPV contains a hydroxy ethylene dipeptide isostere group, whereas RTV holds a longer side isopropyl thiazolyl group for inhibiting CYP3A4 and boosting the circulating concentration of other antiviral PIs [10,185]. On the other hand, DRV has a benzyl group that may hinder off-target access to the host protease. Thus, the next generation of PIs should incorporate fused ring polycyclic ethers and aromatic heterocycles to promote hydrogen bonding interactions with the backbone atoms of aspartyl HIV-1 protease as well as van der Waals interactions with residues in the enzyme internal cavities. DRV was developed with such structural consideration [186] and has been proven to have much less off-target effect than that of LPV or RTV. Another approach for safer PIs is screening a large database of active molecules or designing multiple analogs of current PI drugs through structure-based molecular docking and simulation analysis [187]. An analog of ATV has been screened out with a greater inhibitory capacity on HIV-1 aspartyl protease and less off-target effect/hepatotoxicity than the original ATV drug [188].

## 5. Conclusive Remarks

The factors and molecular pathways underlying PI-associated liver injury are complex and include glucose transporters GLUT2 and GLUT4, organic ion transporters OATP1B1 and OCT1, drug-metabolizing P450 isoenzymes, efflux transporter P-glycoprotein, lipid transporting ApoB, transcription regulators FXR, HNF4α, and PXR, lipogenic regulators C/EBPs, PPARγ, and SREBPs, insulin signaling adapter protein IRS1, AKT/PKB signaling, bilirubin-conjugating UDPGT, redox regulator Nrf2, ROS, and inflammatory cytokines, off-target proteases RCE1 and STE24 and their substrates small GTPase Rab proteins that regulate ER-Golgi trafficking, prolong unfolded protein response and organelle stress response, activation of CHOP, and increase of hepatocellular apoptosis. In addition to the PIs, other general factors could add layers of complexity to HIV drug-associated hepatotoxicity. First, HIV/SARS-CoV2 PIs are metabolized extensively by the liver and have potentially important interactions with other types of antiretroviral agents, alcohol consumption, or substance uses such as cannabinoid and methadone that compete for liver metabolizing enzymes. Second, there are potentially complex interactions of the antiviral protease inhibitors with multimorbidity in an aging population of PLWH who are under life-long ART. Third, PIs could also have interactions with underlying hepatic impairment from ongoing virus infections as both viral infections and anti-viral therapies cause organelle (e.g., ER) stress, and there is a paradox as to whether ER stress/UPR activation should be manipulated for cell survival, which could either reduce drug hepatotoxicity or favor virus replication. Despite these complications, new generations of antiviral protease inhibitor drugs with efficient delivery capacity, high bioavailability, enhanced affinity to viral proteases, and fewer interactions with host off-targets provide promising pharmaceutical solutions to PI-associated liver damages in HIV/AIDS patients.

## Figures and Tables

**Table 1:** Hepatic factors/pathways affected by HIV/SARS-CoV2 protease inhibitors.

Drug Names	Molecular Factors and Pathways	Pathological Consequences
IDV, LPV, NFV, RTV, SQV	GLUT4, IRS1&2, GLUT2, Insulin Signaling	
	AKT/PKA, PKCε, JNK1 ApoB, C/EBPα, PPARγ, SREBP	Insulin Resistance, Dyslipidemia
ATV, NFV, RTV	ROS, UCP2, CYP450, Nrf2, HO1, GST, SREBP	
IDV, NFV, RTV, SQV RTV, SQV ATV, IDV	OCT1 MRP2/ABCC2, OATP1B3 UDPGT1A1, UDPGT1A3, UDPGT1A7	Dysfunction of Transporters, Hyperbilirubinemia
RTV, LPV	FXR, PXR, SREBP, HNF4α, CYP3A4, GLUT2 G0/G1 Arrest, NFκB/Akt Signaling, BAX, BCL2 Caspase 3&8, TNFα, IL-1β, IL-6, IL-10, eNOS	Lipid Accumulation, Cell Death, Immune Dysfunction
RTV, Tipranavir IDV, NFV, Cannabinoids, Alcohol, ATV, DRV, LPV, Azoles SQV, RTV, Rifampicin ETV, RTV, Cobicistat, Statins, Telaprevir RTV, DRV, LPV, MPV, NMV, Amoxicillin, Interferon, Ribavirin	CYP3A	
	CYP3A4	
	CYP45014DM	
	CYP3A4 OATP1B1, P-gp, CYP3A4, CYP3A5	Elevation of ALT and AST, Biliary and Hepatic Injuries
	GST, ACE2, CYP450	
APV, LPV, RTV, DRV, DEX, RDV, EFV, DTG, NFV, Alcohol	UPR, IRE1, ATF6, PERK, CHOP, ER Stress C/EBPβ, CREBP3, TFE3, Rab Proteins Golgi Stress, SREBP, ACC, FAS, SCD1	Cell Death, Inflammation, Fatty Liver, Liver Fibrosis

APV, amprenavir; ATV, atazanavir; DEX, dexamethasone; DRV, darunavir; DTG, dolutegravir; ETV, elvitegravir; IDV, indinavir; LPV, lopinavir; MPV, molnupiravir; NFV, nelfinavir; NMV, nirmatrelvir; RDV, remdesivir; RTV, ritonavir; SQV, saquinavir. ApoB, apolipoprotein B; ACC, acetyl-CoA carboxylase; ACE2, angiotensin-converting enzyme 2 receptor; ALT, alanine transaminase; AST, aspartate transaminase; ATF6, activating transcription factor 6; Bcl2, B-cell lymphoma-2; C/EBP α and β, CCAAT/enhancer- binding protein α and β; CHOP, C/EBP homology protein 10; CREBP3, CAMP responsive element binding protein 3; CYPs, cytochrome P450 enzymes; eNOS, endothelial nitric oxide synthase 3; ER, endoplasmic reticulum; FAS, fatty acid synthase; FXR, farnesoid X receptor; GLUT2&4, glucose transporter 2 and 4; GST, glutathione-S-transferase; HNF4α, hepatocyte nuclear factor 4α; HO1, heme oxygenase-1; IL-1β, 6, and 10, interleukin 1β, 6, and 10; INSTIs, integrase strand transfer inhibitors; IRS1&2, insulin receptor substrate 1 and 2; IRE1, inositol requiring enzyme 1; JNK, c-JUN N-terminal kinase; MRP2/ABCC2, canalicular efflux transporter multidrug resistance-associated protein 2; NFκB, nuclear factor κ B; Nrf2, nuclear erythroid-derived factor 2; OATP1, organic anion transporting polypeptide 1; OCT1, human organic cation transporter 1; PERK, PKR-like ER-localized eIF2α kinase; P-gp, P-glycoprotein; PKA, protein kinase B; PPARγ, peroxisome proliferator-activating receptor γ; PXR, pregnane X receptor; RDV, remdesivir; ROS, reactive oxygen species; SCD1, stearoyl-CoA desaturase 1; SERCA, sarco/endoplamic reticulum Ca2+-ATPase; SREBP, sterol regulatory element binding protein; TFE3, transcription factor for immunoglobulin heavy-chain enhancer 3; TNFα, tumor necrosis factor α; UDPGT, uridine-diphosphoglucuronic glucuronosyltransferase; UPR, unfolded protein response.

## Data Availability

Not applicable.
